# Supplementary material for: Rehoming and Other Refinements and Replacement in Procedures Using Golden Hamsters in SARS-CoV-2 Vaccine Research
Source: Animals (Basel). 2023 Aug 14;13(16):2616. doi: 10.3390/ani13162616 (PMC10451472; doi:10.3390/ani13162616)
Supplement: Supplementary file 1 [file animals-13-02616-s001.zip › animals-2515786-supplementary.pdf]

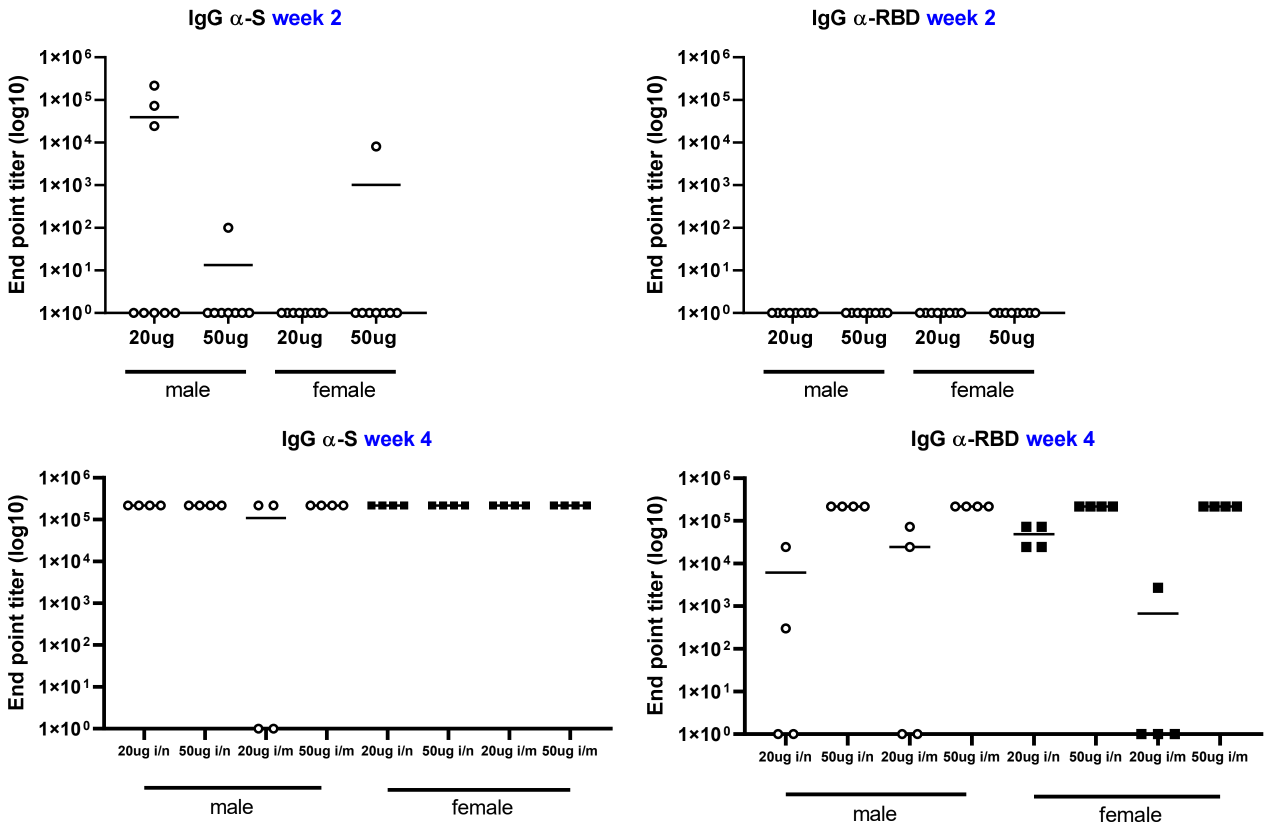

Figure S1: Booster dose after 2 weeks induced high titers of antibody production. Hamsters were immunized with plasmid DNA encoding RBD-bann with two different doses (20 μg or 50 μg) and two modes of adminstration. Graphs represent the mean EPTs of groups of hamsters (n = 4 per group) i/m – intamuscular application, i/n-intranasal. Each dot represents a value of IgG against RBD or Spike in individual animal. ELISA test detected antibodies against RBD and Spike.

To determine specific types of anti-RBD antibodies in the hamster sera, Goat Anti-Syrian Hamster IgG H&L – HRP (Abcam, AB ab6892) were used. Endpoint titre (EPT) was determined as the dilution above the value of the cutoff. The cutoff value was determined from the absorbance data of the control hamster serum (sampled before vaccination and tested negative to natural coronavirus infection). Cutoff for each dilution in ELISA (100, 300, 900, 2700, 8100, 24300, 72900, 218700) was calculated from absorbance based on the equation average + standard deviation*2,177 . For a confidence level of 95%, a value of 2,177 was used as the standard deviation multiplier based on the paper of Frey at el.

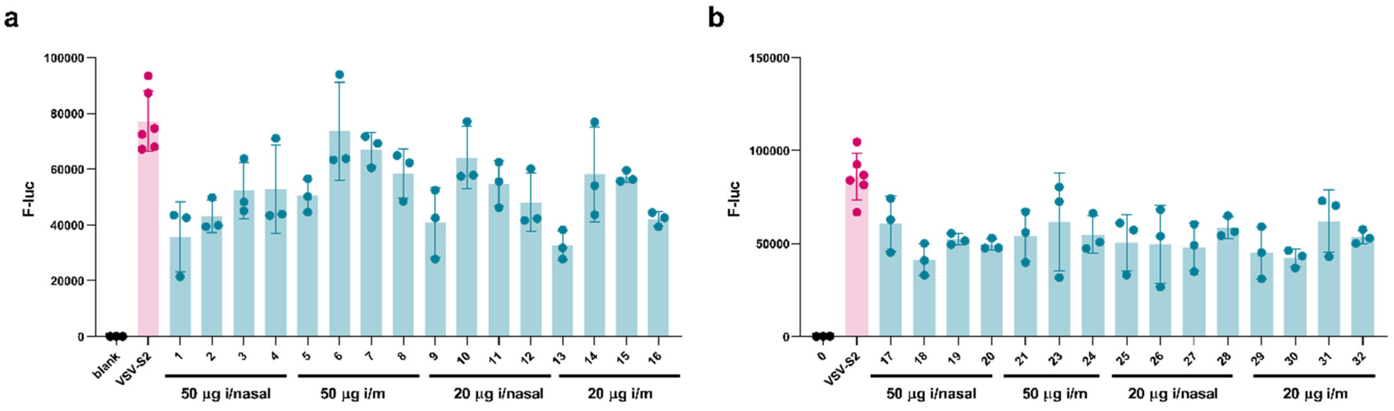

Figure S2. Pseudovirus (PV) neutralisation assay. Sera of hamsters immunized with DNA vaccine encoding scaffolded RBD-bann 2 weeks after the boost (second dose) were diluted 50-fold, and the spike-pseudotyped virus infection of ACE2 and TMPRSS2-transfected HEK293 cells was measured by luminescence. VSV-S2 act as a positive control of HEK293 cell infection. Each column represents one serum sample, biological replicates of HEK293 cell infections are shown, panel a) for females and b) for males, grouped by quantity of vaccine candidate given (20 or 50 µg per animal at each dose) or mode of inoculation (i.m. or i.n.), Values as close to zero (blank) would represent effective neutralisation.

Immunized hamster sera were pre-incubated with the spike pseudovirus for 30 min before addition to the cells. The next day, the medium was removed, and the cells were lysed in a passive lysis buffer Biotium, Fremont, CA, USA. Firefly luciferase activity as a measure of PV infection and coelenterazine H (Xenogen) to follow the Renilla luciferase activity for the determination of transfection efficiency and normalization was used to monitor the state of PV neutralisation.

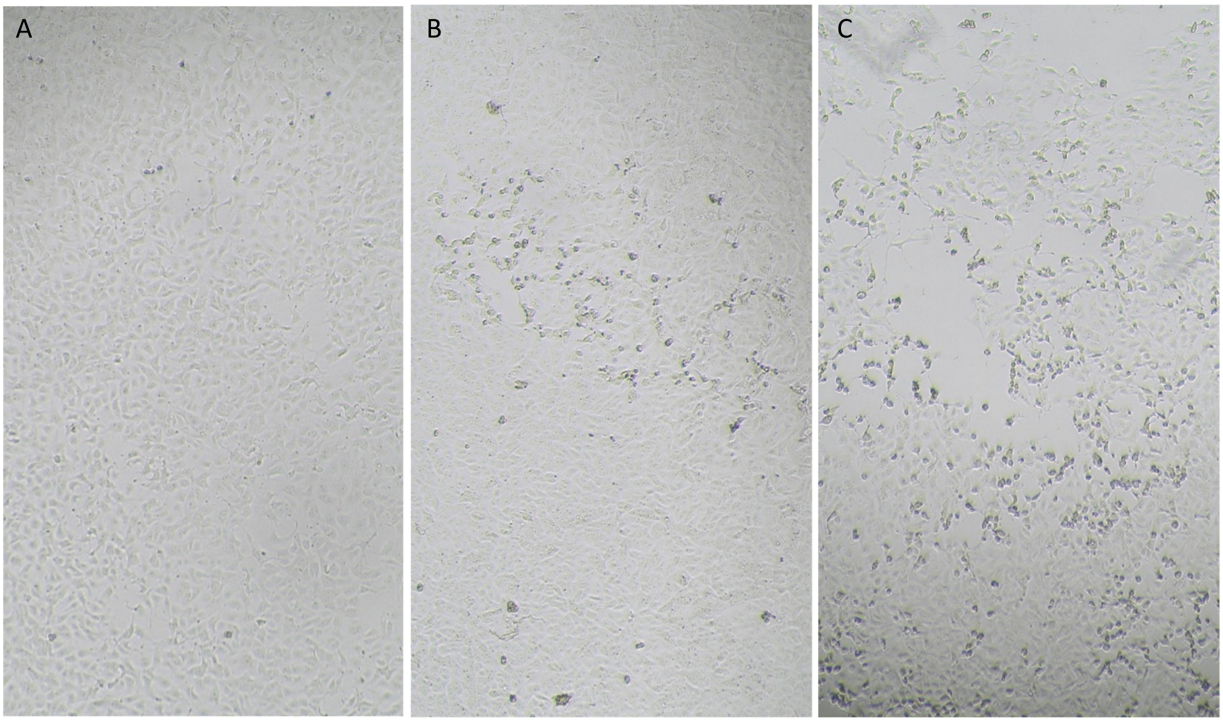

Figure S3: Microphotographs of Vero 6 cells in VNT test. A: intact, control medium after 5 days (dpi), B: CPE 5 dpi, C: another example of CPE 5 dpi.

VNT cut off is detectable CPE in Vero E6 monolayer after incubation of 110 h, observed by inverted light microscope at e.g. 400-fold magnification. Characteristic morphological changes in cells (rounding of adherent infected cells) in the culture that were the target of SARS-CoV-2 replication were labelled as CPE.

The number of infectious viral particles was quantified using the Median Tissue Culture Infectious Dose ($TCID_{50}$) test. The assay is based on adding a serial dilution of the defined virus to the susceptible cells in 96-well plate. The dilution at which 50% of the wells show CPU is used to mathematically calculate the $TCID_{50}$ of the virus sample as generally described.

Table S1. Pilot viral challenge with intranasal inoculation of 25 µl 5×10⁴ TCID₅₀/mL SARS-CoV-2 strain Slovenia/ SI -4265/20 to determine endpoint criteria and best sampling tissue.

25 µl 5×10^4 $TCID_{50}$/ml intra nasal inoculation			
day of sacrifice	2 dpi (n=2)	4 dpi (n=2)	7 dpi (n=2)
body mass change	2,30%	-5%	-1%
	RT-qPCR - viral RNA detection (Ct average)		
oropharynx	detected (18,8)	detected (23,6)	detected (26,3)
caudal nasal cavity	detected (21,8)	detected (17,3)	detected (21,5)
lung - tissue	not detected	detected (26,0)	detected (36,1)
trachea – mucosal swab	not detected	not detected	not detected
duodenum – mucosal swab	detected (35,2)	minimal (nd/35,0)	detected (34,7)

Pilot viral challenge was repeated with increased dose, animals was killed on day 4 and only lung tissue sampled (see Table 1 in the main text and Figure S4 below).

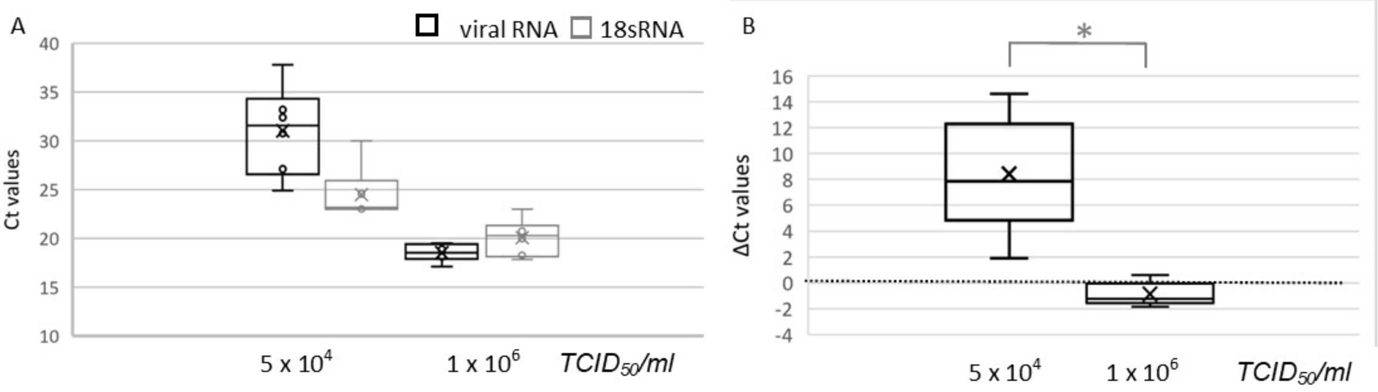

Figure S4. RT-qPCR results from lung tissue homogenates. A) Ct values for RNA viral loads of two different infectious doses tested. 18sRNA was used as an endogenous control. B) Difference between RNA viral load expressed as ΔCt. *significant at p=0,002. Outliers were identified by IQR method.

The real-time RT-PCR test was used as a qualitative test. All samples with the fluorescent signal increases exponentially and, producing an exponential curve were treated as positive. All samples were tested with the same procedure. In this study, the LOD was not performed.

To detect endogenous control, we used TaqMan Gene Expression Assays from Applied Biosystems (Thermo Fisher Scientific), the assay ID Hs99999901_s1 (for 18S rRNA). For all RT-qPCR assays, AgPath-IDTM One-Step RT - qPCR Reagents (Thermo Fisher Scientific) were used. Thermal cycling for all assays was performed at 45°C for 10 min for reverse transcription, followed by 95°C for 10 min and then 45 cycles of 95°C for 15ns and 58°C for 45 s.

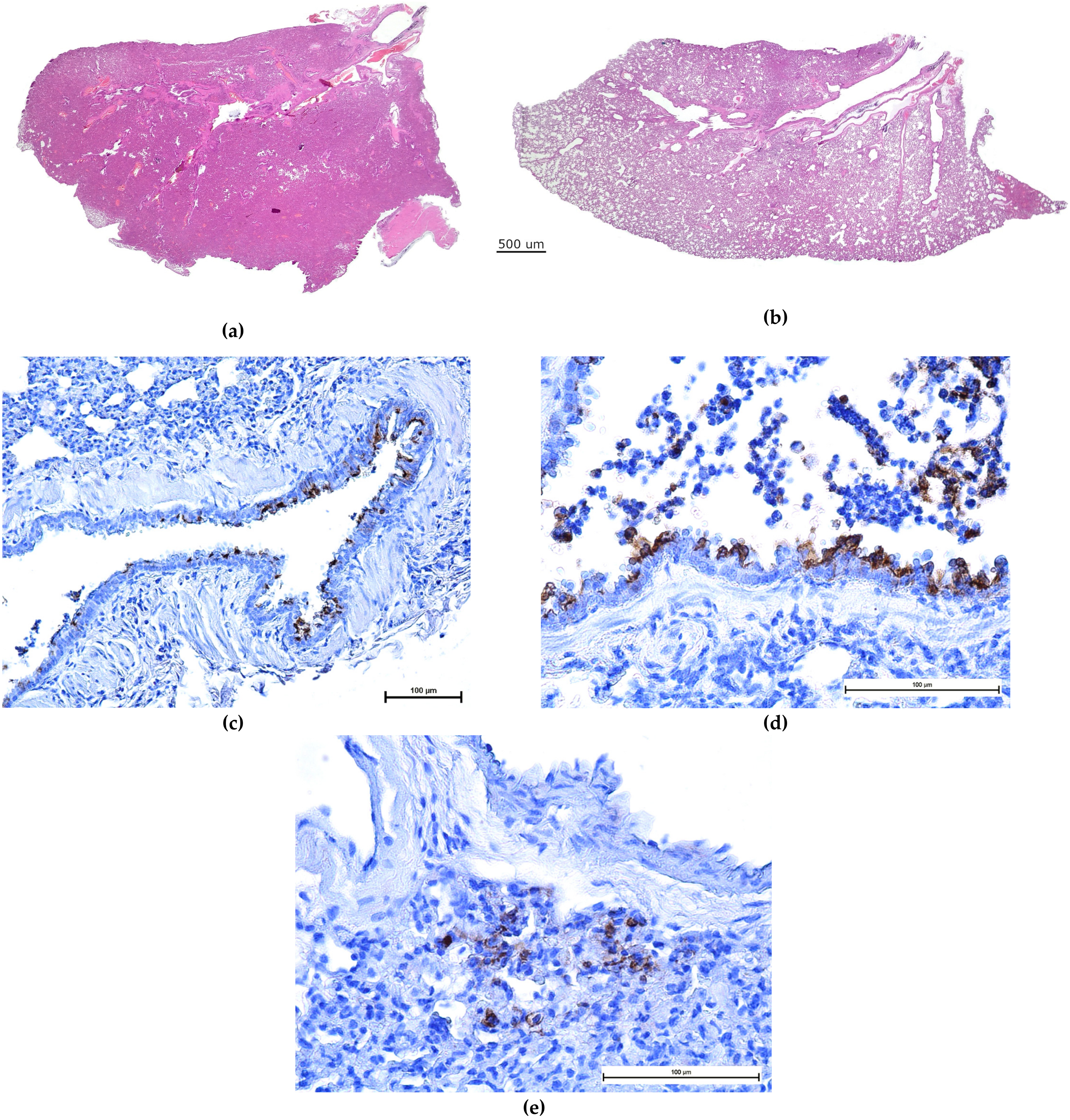

Figure S5: Lung histology: a) cross section of whole left lung lobe in infected animal, HE stain, day 4 p.i..wide atelectasis is seen, due to pathological changes (not scored at this stage of the project); b) normal, noninfected animal, HE; c-e Immunohistochemistry, spike protein *in situ* on day 4 p.i.- brown precipitate, c) lobar and segmental bronchi 200x magnification, d) 400 x magnification of viral particles in respiratory epithelium and in exudate with macrophages, e) few interstitial foci as found in 2 individuals only (scored ++ in Table 2).

Immunohistochemistry procedure: Paraffin embedded lung was cut at 5 μm horizontal sections and placed on electrostatically treated microscope slides (Superfrost Plus, Fischerbrand). For immunostaining, a Novolink Polymer Detection System kit (Leica Biosystems, Newcastle, UK) was used according to the manufacturer's instructions, with antigen retrieval step (5 minutes of low power microwave heating in citrate buffer pH 6,0) and Tween-PBS as rinsing buffer. SARS-CoV-1/2 Spike Protein (2B3E5) mouse mAb were used (#52342, Cell Signaling Technology, Danvers, Massachusetts, USA) at a dilution of 1:200 and an incubation time of 2 hours (humified chamber at RT) as primary antibodies. Secondary antibodies were left incubated for 1 hour at RT. One slide per animal was also HE stained (Leica Autostainer) for planned pathology assessment. After staining slides were dehydrated by immersion in increasing concentrations of ethanol, cleared with Neoclear (Merck, Darmstadt, Germany), and cover slipped. Images of slides were captured and analysed with NIS Elements BR v4.6 (Nikon) software on Nikon Eclipse Ni microscope with Nikon DS-Fi1 camera. Whole slide (HE) photograph was taken with stereomicroscope Zeiss Stemi 508, Zeiss Axiocam 208 and Zeiss ZEN 3.1 (blue ed.) software.

Translation of Anonymous survey via local platform www.enka.si -

Text by email:

Last year you adopted a hamster that donated blood for science to test COVID -19 vaccine candidates and was no longer needed - for which I thank you again. I will soon be presenting the good practice of the"rehoming" procedure at the Slovenian Congress of Laboratory Animal Science. I would like to collect as much feedback as possible from adopters. I kindly ask you to fill in the (anonymous) survey: https://www.1ka.si/admin/survey/index.php?anketa=416241

Please answer the following questions, the results will be anonymous.

Q1

Were you generally satisfied with the animal adoption?

Yes / No / Yes in some respects

Other:

Q2

Have you left the animal in the care of another person for a long time or permanently?

Yes / No

Q3

How long the animal lived/still lives; approx. in months?

Q4

Mark your agreement with the statements

The hamster has adapted well to the new environment

The hamster accepted various environmental enrichments and new types of food

The hamster was looking for interaction with a human, it was not hiding

The hamster was healthy

I could easily decide or recommend on a similar adoption of a laboratory animal in the future

Q5

Adoption of surplus or retired animals that have been bred or used in procedures for scientific purposes can improve public attitudes toward the use of laboratory animals and help restore general confidence in science.

I agree

Not likely

Can’t tell

Q6

Do you have any other comments, advice or wishes? Your response will be confidential.
